# IntestLine: a shiny-based application to map the rolled intestinal tissue onto a line

**DOI:** 10.1093/bioinformatics/btad140

**Published:** 2023-03-21

**Authors:** Altay Yuzeir, David Alejandro Bejarano, Stephan Grein, Jan Hasenauer, Andreas Schlitzer, Jiangyan Yu

**Affiliations:** Quantitative Systems Biology, Life & Medical Sciences (LIMES) Institute, University of Bonn, Bonn 53115, Germany; Quantitative Systems Biology, Life & Medical Sciences (LIMES) Institute, University of Bonn, Bonn 53115, Germany; Faculty of Mathematics and Natural Sciences, University of Bonn, Bonn 53115, Germany; Faculty of Mathematics and Natural Sciences, University of Bonn, Bonn 53115, Germany; Institute for Computational Biology, Helmholtz Center Munich—German Research Center for Environmental Health, Neuherberg 85764, Germany; Quantitative Systems Biology, Life & Medical Sciences (LIMES) Institute, University of Bonn, Bonn 53115, Germany; Quantitative Systems Biology, Life & Medical Sciences (LIMES) Institute, University of Bonn, Bonn 53115, Germany

## Abstract

**Summary:**

To allow the comprehensive histological analysis of the whole intestine, it is often rolled to a spiral before imaging. This Swiss-rolling technique facilitates robust experimental procedures, but it limits the possibilities to comprehend changes along the intestine. Here, we present IntestLine, a Shiny-based open-source application for processing imaging data of (rolled) intestinal tissues and subsequent mapping onto a line. The visualization of the mapped data facilitates the assessment of the whole intestine in both proximal–distal and serosa-luminal axis, and enables the observation of location-specific cell types and markers. Accordingly, IntestLine can serve as a tool to characterize the intestine in multi-modal imaging studies.

**Availability and implementation:**

Source code can be found at Zenodo (https://doi.org/10.5281/zenodo.7081864) and GitHub (https://github.com/SchlitzerLab/IntestLine).

## 1 Introduction

A comprehensive assessment of large organs is a key challenge in many biomedical research fields. Therefore, different strategies have been devised to reduce the spatial field of view for spatial analysis. A commonly used approach for the intestine research is the Swiss-rolling technique, which has been shown to facilitate the study of intestinal structure along the proximal–distal axis in a single image (Bialkowska *et al*., 2016). Yet, the visualization of the imaging data for the intestinal tissue as an artificial role is highly nonintuitive and limits the comprehension.

**Figure 1.**
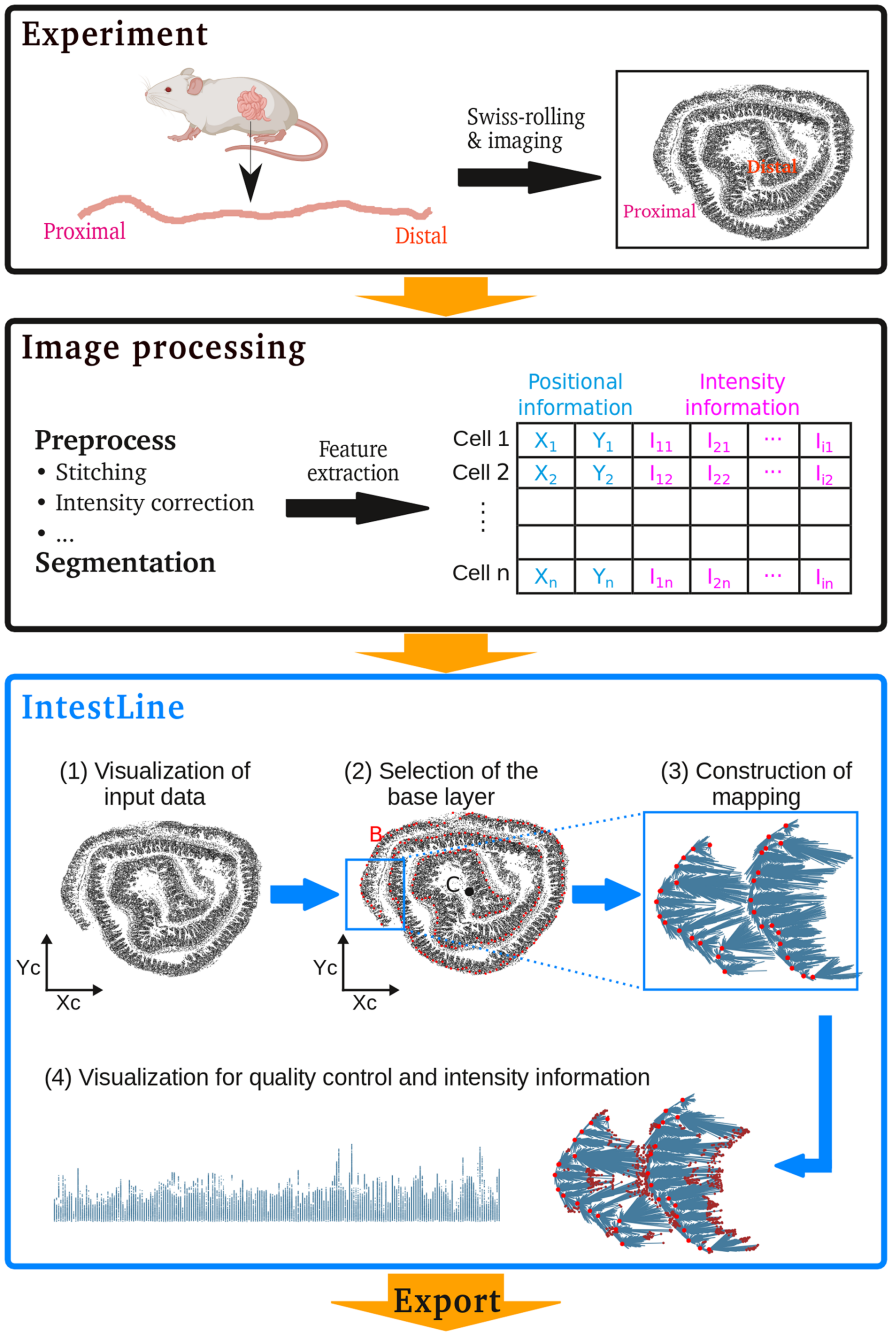
Workflow of IntestLine. Experimental data for rolled intestine is preprocessed using standard imaging pipelines to obtain a csv-file containing information about position and intensity. (1) This input is used to visualize the location of individual cells. (2) The user can manually select the center (C) of the Swiss-roll, and the points forming the base layer (B) using the built-in Shiny app. (3) Cells are mapped to nearest outer adjacent base layer point to determine position along proximal-distal axis and distance from base layer. (4) Visualizations are provided for the assessment of the mapping as well as the study of the tissue slice. For the latter, a linearized view of the intestine is created. All obtained information can be exported for further study

Here, we present IntestLine, a Shiny-based tool to process images of rolled intestinal tissue (Fig. 1). The IntestLine pipeline uses the x–y position of segmented cells as the starting point and implements four major steps: (i) visualization of cell positions and their properties; (ii) selection of the base layer (which will act as a skeleton for the reconstruction) and ordering of base layer points from proximal to distal; (iii) assignment of all cells to the closest proxy on the base layer; and (iv) visualization of the intestine in a linear coordinate system using the positioning implied by the coordinate on the base layer. Furthermore, IntestLine allows for the export of the mapped positional information for subsequent analysis and visualization on other tools.

## 2 Features

In the following, we provide a brief description of the features of IntestLine. A detailed description of the method is provided in the Supplementary material.

### 2.1 Input format

IntestLine is tailored for the analysis of multi-modal imaging data of rolled intestinal tissues obtained via the co-detection by indexing (CODEX) system (Hickey *et al*., 2021), 10x Visium spatial gene expression pipeline (Parigi *et al*., 2022) and related approaches. For the processing and segmentation of these images, a series of pipelines is available (Stirling *et al*., 2021; Lee *et al*., 2022). IntestLine builds on the results from these imaging process pipelines and uses a csv-file with x- and y-positions of individuals cells as well as their (corrected) intensity as input.

### 2.2 Processing and map construction

IntestLine supports the construction of mappings from the original image of the rolled tissue to alternative representation which preserves (i) the ordering along the proximal–distal axis and (ii) the distance of cells from the base layer. Upon uploading the csv-file, IntestLine provides a visualization of the positional information. Within this visualization, users can select points along the base layer, which is from the inner (distal) to outer (proximal) side of the image, indicating the boundaries between spiral revolutions. The number of used points can be chosen by the user to allow for a problem-specific resolution.

IntestLine uses the selected points on the base layer to construct a representation of the intestine. Therefore, for each cell, the corresponding base-layer point is determined by a directionally biased nearest neighbor analysis. This nearest neighbor search considers only the base layer in the outward direction to preserve information about the thickness of the intestinal wall and the position of cells within the wall.

IntestLine supports the removing of cells that are noisy assignment based. Cumulative histogram curves of quality control scores are provided within the pipeline, allowing for user-defined filtering thresholds.

### 2.3 Visualization

IntestLine provides visualizations for the assessment of the constructed mapping, e.g. the assignment of cells to points on the base layer. This can be used to guide the refinement of the selection of points in the base layer, as well as to pinpoint problems in the images (e.g. base-layer ruptures).

For the visualization of the experimental data, IntestLine allows for the visualization of the unrolled data. This depicts the cells (and their intensities) along the proximal-distal axis (x-coordinate) with their distance from the base layer (y-coordinate). Cells can be color-coded according to intensity values provided in the input csv-file. An example to visualize fluorescent marker intensity derived from CODEX system is shown in the Supplementary material.

### 2.4 Export

IntestLine supports the export of all images as png-files. Furthermore, the selected points on the base layer can be exported and re-uploaded into the application for future analysis. In addition, the mapping results containing mapped linear coordinates and statistical scores for the mapping, together with original parameters from the input file, can be exported as a csv-file for other analysis.

## 3 Implementation and availability

IntestLine is an open-source application and available as a docker image under the BSD-3 clause License. The source code is deposited in Zenodo (https://doi.org/10.5281/zenodo.7081864) and GitHub (https://github.com/SchlitzerLab/IntestLine). The vignette and the tutorial video to use IntestLine can be found in the GitHub repository.

## 4 Conclusion

The processing of imaging data is key for the assessment of biological processes. Here, we have presented IntestLine, the first open-source application to unroll images of rolled intestinal tissue. Using an image obtained from the CODEX system, we have shown that IntestLine could provide a high-resolution mapping of the rolled intestinal tissue on a linear coordinate system. The availability of the tool allows others in the community to contribute, e.g. to the integration of IntestLine to image processing pipelines, and to the visualization capabilities of IntestLine itself.

## Supplementary Material

btad140_Supplementary_Data
