# Supplementary material for: IntestLine: a shiny-based application to map the rolled intestinal tissue onto a line
Source: Bioinformatics. 2023 Mar 21;39(4):btad140. doi: 10.1093/bioinformatics/btad140 (PMC10081871; doi:10.1093/bioinformatics/btad140)
Supplement: btad140_Supplementary_Data [file btad140_supplementary_data.docx]

**Supplemental Information**

IntestLine: a Shiny-based application to map the rolled intestinal tissue onto a line

Altay Yuzeir^1^, David Alejandro Bejarano^1^, Stephan Grein^2^, Jan Hasenauer^2,3,*^, Andreas Schlitzer^1,*^, Jiangyan Yu^1,*^

^1^Quantitative Systems Biology, Life & Medical Sciences (LIMES) Institute, University of Bonn, 53115 Bonn, Germany, ^2^Faculty of Mathematics and Natural Sciences, University of Bonn, 53115 Bonn, Germany, ^3^Institute for Computational Biology, Helmholtz Center Munich – German Research Center for Environmental Health, 85764 Neuherberg, Germany

*To whom correspondence should be addressed.

Contact: jiangyan.yu@uni-bonn.de, andreas.schlitzer@uni-bonn.de or jan.hasenauer@uni-bonn.de

**Material and** **Methods**

IntestLine is an open-source application and is implemented in a docker with the pre-installed Shiny app. To map the rolled intestinal tissue on a line, IntestLine consists of four major steps:

1) To create linear visualization of the intestine from the images of a slice prepared by the Swiss-rolling method, users first upload a csv-file containing cell locations to the IntestLine application. In order to assign all cells to the base layer, a center point needs to be selected by the user.

2) Next the dataset will be uploaded into the Shiny app to allow users to select points for the base layer. IntestLine makes use of the order of points picked in the Shiny app to reconstruct the base layer of the linear coordinate system and thus it is important to select points from inner (distal) to outer (proximal) in order. The selected points for base layer could be downloaded as a csv-file and be re-uploaded for future analysis.

3) After that, each spot (or cell) in the image will be assigned to the nearest base layer point, which has a larger distance to the center point than the query spot. Meanwhile the distance of the spot to the corresponding base layer point is calculated and will be later used as the thickness (as y-axis) in the linear coordinate system.

4) Later, in order to remove noisy signals in the gap of two intestinal layers, for the group of spots assigned to the same base layer point, the Z-score of distances within the group of spots will be calculated. Users can define their own threshold on the thickness and the Z-score in the filtering step within the app. Finally, the rolled image will be converted into a linear coordinate system using thickness as y-axis, and the cumulative length of base layer points as x-axis. The linear mapping can be exported for further visualization and analysis.

**Example to process an image generated from the CODEX system**

To evaluate IntestLine, we consider a CODEX image of the murine intestine that was prepared by Swiss-rolling technique and was stained with a 15-plex antibody panel. The resulting image was segmented using the CODEX processor V1.7, yielding a total of 150,793 cells (**Fig. S1A**). First, we uploaded the file containing cell locations (xy-coordinates) exported from the CODEX processor into the IntestLine application. Next, we manually selected a base layer containing 1,059 points (**Fig. S1B**). After assigning cells to the base layer (**Fig. S1C**), we performed a stringent filtering by removing noisy assignment with thickness >1,000 or Z-score >2 (**Fig. S1D-E**). As a result, more than 93% of cells were successfully assigned to the outer adjacent base layer (**Fig. S1F**). Finally, we visualized the image in a linear coordinate system as shown in **Fig. S1G**. The mapping of the intestine on a line allows us to observe clear thickness differences in proximal-distal axis. The results show that IntestLine is able to provide a high-resolution mapping of an image of a rolled intestinal tissue to a line.

**Capabilities of converted linear coordinate system to visualize fluorescent marker intensity**

To demonstrate the importance of the data provided by IntestLine, we compared the fluorescent intensities of several markers between the original coordinate system and the converted linear coordinate system. While the direct visualization of the data does not facilitate the identification and quantification of trends, the unrolling provides a clear picture of the change along the serosal-luminal axis of the tissue (**Fig. S2**). For example, Villin staining clearly highlighted the epithelium on top of the structure, whereas olfactomedin-4 (Olfm4) and Ki67 staining accurately revealed the location of proliferating intestinal stem cells at the base of the crypts of the murine small intestine. Meanwhile, lysozyme staining labeled Paneth cells at the base of distal intestinal region. Moreover, the linear representation within the intestinal wall allowed the identification of regions with abnormal marker expression levels. For instance, the region highlighted in **Fig. S2B** is with low expression of Ki67 and lysozyme, suggesting a disrupted structure in this neighbourhood. In comparison, this information is less obvious from the intestinal image in the spiral shape. Taken together, the linear coordinate system would allow better visualization in both proximal-distal and serosa-luminal axes of the whole intestinal structure.

**Supplementary Figures**

**
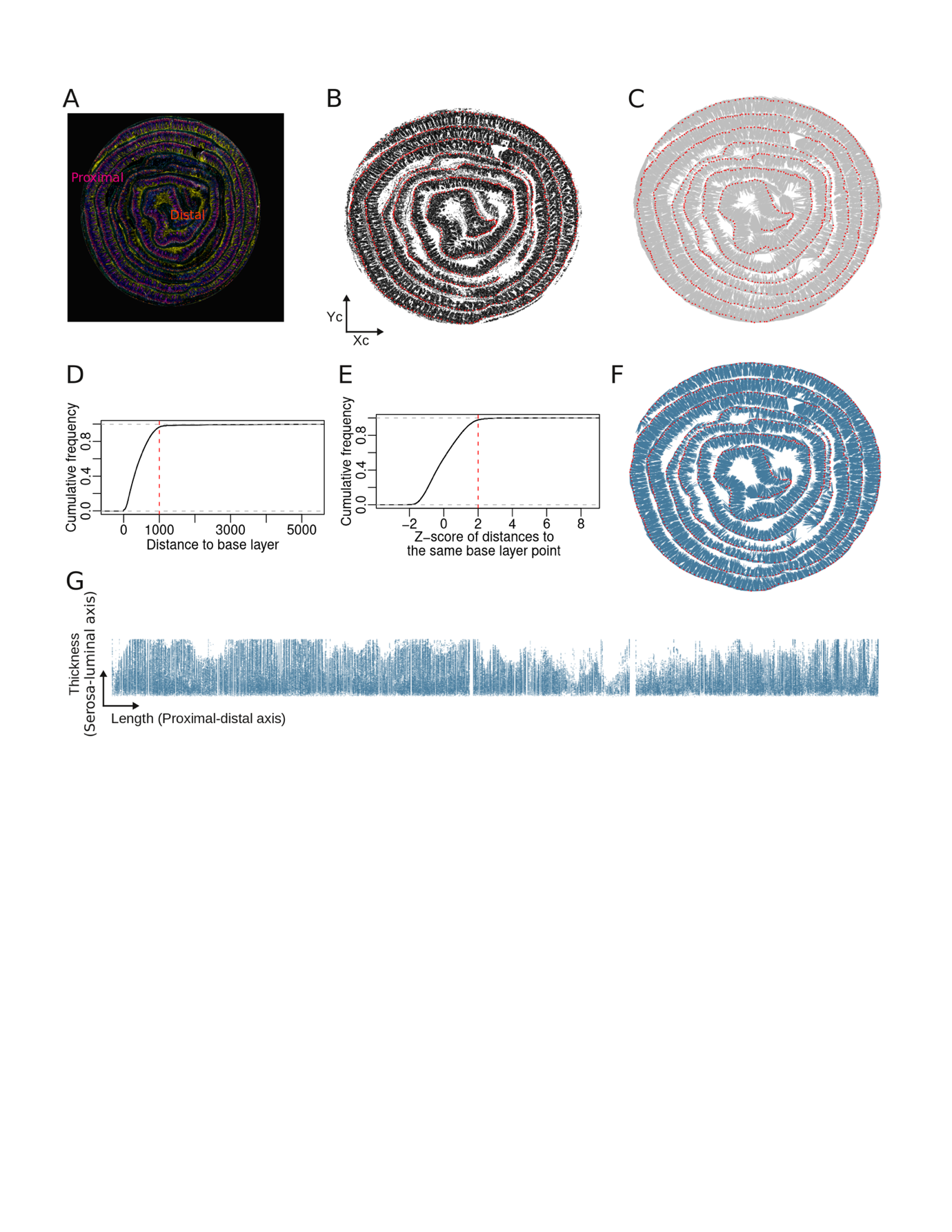
**

**Fig. S1**. Pilot study of mapping a rolled intestinal tissue onto a line. (A) The rolled intestinal tissue imaged by the CODEX technique. (B) Manually selected base layer (depicted in red) for the image. (C) Visualization of assigning spots to base layer before filtering process. The grey line connects the cell or spot to its corresponding base layer point (denoted in red). (D) Cumulative distribution of the thickness (distance of the spot to its corresponding base layer point). (E) Cumulative distribution of Z-score of distances within a group of spots assigned to the same base layer point. (F) Visualization of the successful assignment after filtering out assignment with thickness > 1,000 or Z-score > 2. (G) Converted linear coordinate system of the tissue. Thickness (y-axis) is the distance of the spot to its corresponding base layer point. Length (x-axis) is the cumulative length of base layer points.

**
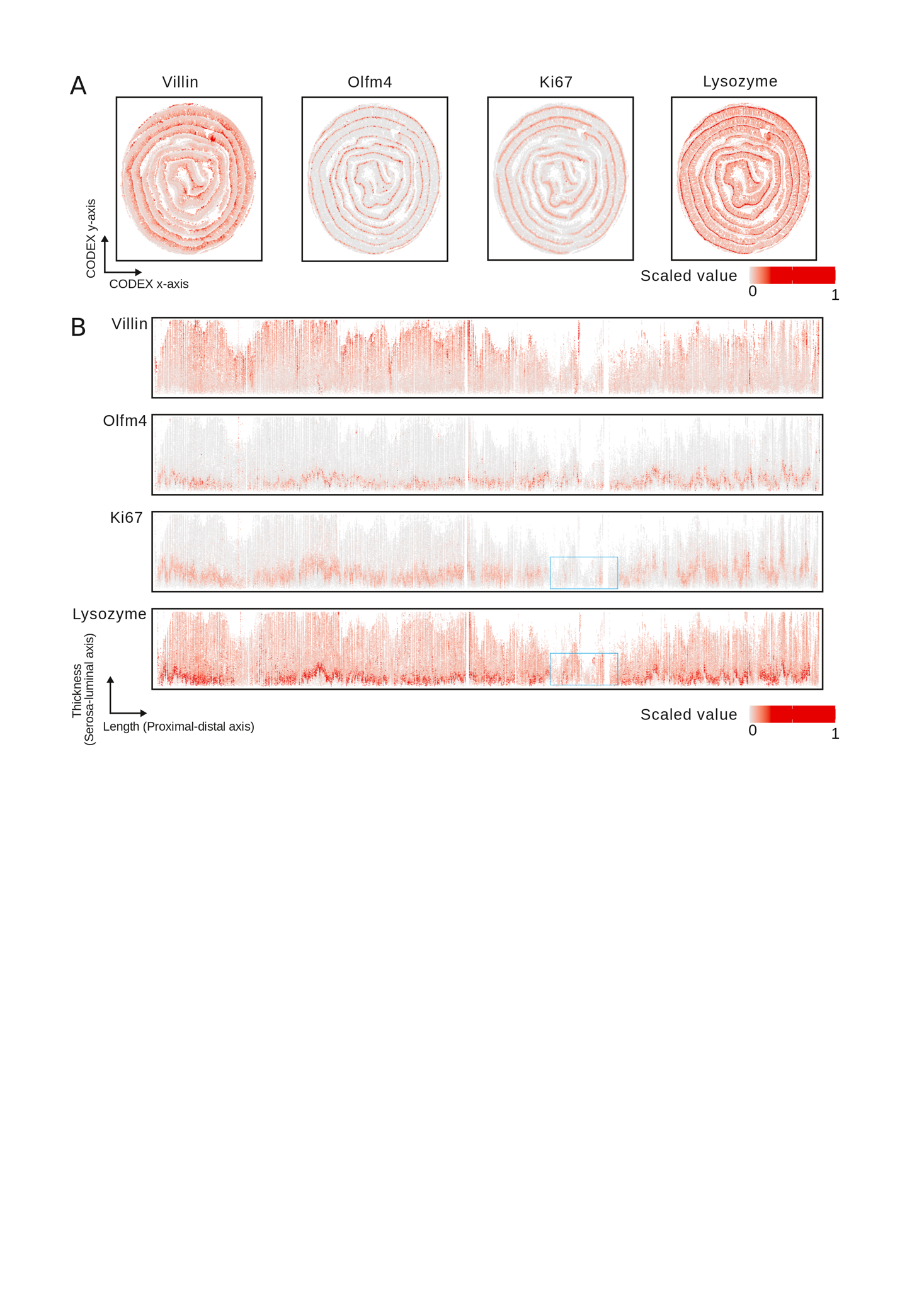
**

**Fig. S2**. Visualization of fluorescent marker intensity on the tissue. (A) Fluorescent signal intensities of Villin, Olfm4 (intestinal stem cell marker), Ki67 (proliferation marker) and lysozyme in original CODEX xy-coordinates. (B) Fluorescent signal intensities of Villin, Olfm4, Ki67 and lysozyme in the converted linear coordinate system. The region lacking of Ki67 and lysozyme expression is highlighted in a blue box.
